# Pulmonary vascular dysfunction in ARDS

**DOI:** 10.1186/s13613-014-0028-6

**Published:** 2014-08-22

**Authors:** Donal Ryan, Stephen Frohlich, Paul McLoughlin

**Affiliations:** 1 Department of Anaesthesia and Intensive Care Medicine, St Vincent’s University Hospital, Elm Park, Dublin 4, Ireland; 2 Conway Institute of Biomolecular and Biomedical Science, School of Medicine and Medical Science, University College Dublin, Belfield, Dublin 4, Ireland

**Keywords:** ARDS, Pulmonary haemodynamics, Pulmonary vascular resistance, Pulmonary vascular dysfunction, Acute cor pulmonale, Outcome

## Abstract

Acute respiratory distress syndrome (ARDS) is characterised by diffuse alveolar damage and is frequently complicated by pulmonary hypertension (PH). Multiple factors may contribute to the development of PH in this setting. In this review, we report the results of a systematic search of the available peer-reviewed literature for papers that measured indices of pulmonary haemodynamics in patients with ARDS and reported on mortality in the period 1977 to 2010. There were marked differences between studies, with some reporting strong associations between elevated pulmonary arterial pressure or elevated pulmonary vascular resistance and mortality, whereas others found no such association. In order to discuss the potential reasons for these discrepancies, we review the physiological concepts underlying the measurement of pulmonary haemodynamics and highlight key differences between the concepts of resistance in the pulmonary and systemic circulations. We consider the factors that influence pulmonary arterial pressure, both in normal lungs and in the presence of ARDS, including the important effects of mechanical ventilation. Pulmonary arterial pressure, pulmonary vascular resistance and transpulmonary gradient (TPG) depend not alone on the intrinsic properties of the pulmonary vascular bed but are also strongly influenced by cardiac output, airway pressures and lung volumes. The great variability in management strategies within and between studies means that no unified analysis of these papers was possible. Uniquely, Bull et al. (Am J Respir Crit Care Med 182:1123–1128, 2010) have recently reported that elevated pulmonary vascular resistance (PVR) and TPG were independently associated with increased mortality in ARDS, in a large trial with protocol-defined management strategies and using lung-protective ventilation. We then considered the existing literature to determine whether the relationship between PVR/TPG and outcome might be causal. Although we could identify potential mechanisms for such a link, the existing evidence does not allow firm conclusions to be drawn. Nonetheless, abnormally elevated PVR/TPG may provide a useful index of disease severity and progression. Further studies are required to understand the role and importance of pulmonary vascular dysfunction in ARDS in the era of lung-protective ventilation.

## Review

### Introduction

Acute respiratory distress syndrome (ARDS) is characterised by diffuse alveolar damage and is frequently complicated by pulmonary hypertension [1]. The single biggest advance in the management of ARDS has been the institution of lung protective ventilation (ARDSNet) [2]. However, mortality remains unacceptably high, ranging from the 32% to 41% reported in randomised controlled trials up to 44% in published observational studies [3],[4].

Intensivists and researchers have long been aware of the occurrence of pulmonary hypertension and cor pulmonale in ARDS. However, there has been uncertainty about the underlying pathophysiology and the link between the degree of pulmonary hypertension and outcome from ARDS. Is pulmonary hypertension simply an indicator of the severity of lung injury or is it part of the underlying pathophysiological process contributing to the development of ARDS? Recent studies have pointed to the importance of pulmonary vascular dysfunction (PVD) in predicting mortality from ARDS [5], but the exact mechanism by which PVD and mortality are linked is not known.

The focus of this review is to examine the nature of the relationship between pulmonary hypertension/PVD and mortality in ARDS.

### Search strategy

Studies were identified after a literature search using key terms (ARDS or acute respiratory distress or ALI or acute lung injury) together with any of the following: pulmonary haemodynamics, pulmonary artery pressure, pulmonary vascular resistance, pulmonary vascular dysfunction, right ventricle, right ventricular failure, acute cor pulmonale, or pulmonary artery catheter. The references of articles found in this manner were also examined for similar studies. Manuscripts that reported a relationship between pulmonary haemodynamics and mortality in ARDS/ALI were included. In addition, papers that reported a relationship between right ventricular failure/right ventricular dysfunction and outcome were included. We have included definitions of commonly used terms in this article in Table 1.

**Table 1:** Definitions of terms used in this article

**Terms**	**Definition**
Pulmonary hypertension (PH)	Mean pulmonary artery pressure (mPAP) >25 mmHg
	Moderate PH - mPAP between 30 and 45 mmHg
	Severe PH - mPAP > 45 mmHg [6],[7]
Pulmonary vascular resistance (PVR)	mPAP-PAOP/cardiac output
Pulmonary vascular resistance index	mPAP-PAOP/cardiac index
Pulmonary vascular dysfunction (PVD)	Abnormal elevations in PVR identified by measurement of either transpulmonary gradient (mPAP-PAOP) and/or pulmonary vascular resistance index (mPAP-PAOP/cardiac index) [5]
Right ventricular dysfunction (RVD)	Pulmonary artery catheter-based definitions:
	(i) RVD = CVP > PAOP and MPAP > 25 mmHg and SVI < 30 ml m^−2^, or
	(ii) RAP > PAOP
Acute cor pulmonale (defined by echo)	Ratio of RV to LV end-diastolic area >0.6 with interventricular septal flattening at end-systole

#### Assessment of pulmonary haemodynamics in ARDS

Many indices of pulmonary haemodynamics have been measured in patients with ARDS. Pulmonary arterial pressure, wedge pressure and pulmonary vascular resistance have all been reported as well as measures of right ventricular function. The two most commonly reported measures are pulmonary arterial pressure and pulmonary vascular resistance.

### Pulmonary arterial pressure and ARDS

A number of studies (Table 2) have documented the changes in pulmonary haemodynamic measurements in patients with ARDS. All measurements were derived from the use of pulmonary artery catheter except for the study by Cepkova [8], where PA systolic pressures were estimated using echo. Some of these studies are small, and the majority were conducted before the widespread introduction of low tidal volume ventilation. Nevertheless, certain observations can be made from the data.

**Table 2:** Studies that relate pulmonary haemodynamic variables to outcome from ARDS

	**Study period**	**Number**	**PAP mmHg (mean PAP unless specified)**	**PVRI wood units (m**^**−2**^**)**	**PAOP mmHg**	**Independent predictors of survival**	
						**PAP**	**PVR (I)**
Zapol and Snider [9]	Pre 1977	30	28 to 32	(2.5 to 4.8)	n/a	N	Y (trend)
Villar et al. [10]	1983 to 1986	30	27 to 28 ± 4 to 7	4.5 ± 1.69 to 5.7 ± 2.06	10 ± 4 to 11 ± 5	N	Y
Squara et al. [6]	1985 to 1987	*586*	26 ± 8.5	3.21 ± 1.75	11.7 ± 4.5	Y	N
Suchyta et al. [11]	1987 to 1990	162	26 ± 8	n/a	n/a	N	-
Hemmila et al. [12]	1989 to 2003	255	Systolic 46 ± 13.5	n/a	17.6 ± 5	N	-
			Diastolic 28.5 ± 8.9				
Osman et al. [13]	1999 to 2001	145	28 ± 8	4.5 ± 2.4	12 ± 5	Y	N
Cepkova et al. [8]	2004 to 2006	42	Systolic 42 ± 9			N	
			Echo derived				
Beiderlinden et al. [14]	Pub 2006	95	35.4 ± 8.8	4.625 ± 2.04	16 ± 5.4	N	-
Bull et al. [5]	2000 to 2005	*501*	31.6 ± 8.3	3.825 (2.49 to 6.48)	17.13 ± 5	N	Y

All haemodynamic data was derived from pulmonary artery catheter use unless otherwise stated. mPAP, mean pulmonary artery pressure; PVRI, pulmonary vascular resistance index; PAOP, pulmonary artery occlusion pressure; PAP, pulmonary artery pressure. Wood units (mmHg/L/min) are multiplied by 80 to convert to standard metric units (dynes.sec.cm^−5^). Normal values for PVR range from 0.3 to 1.6 Wood unit.

Mild to moderate elevations in mean pulmonary artery pressure (mPAP) are seen in most patients with ARDS [15],[16]. Squara et al. found moderate elevation in mean pulmonary pressure in 526 patients, 48 h after the diagnosis of ARDS [6]. Patients with worse PaO_2_/FiO_2_ ratios had higher mPAP than those with better oxygenation (27.9 ± 8.1 vs. 22.3 ± 6.5 mmHg, *p* = 0.0001). Systolic pulmonary arterial pressure (PAP) was deemed to be of ‘independent and sustained prognostic significance during the course of ARDS’. In a later study, Osman et al. also found mPAP to be an independent predictor of mortality in a multivariate model [13]. Other studies either found PAP not to be predictive of death or else did not specifically examine for a relationship [5],[9]–[12],[14].

In patients with severe ARDS, Beiderlinden et al. [14] found an incidence of pulmonary hypertension of 92.2% but did not find any association between pulmonary hypertension and death. Hemilla et al., in a review of patients with severe ARDS who subsequently received ECMO, found evidence of moderate pulmonary hypertension using pulmonary artery catheter data acquired prior to the institution of extracorporeal support [12]. Again, direct measurements of PAP were not identified as being of prognostic significance.

### Pulmonary vascular resistance and ARDS

Pulmonary vascular resistance (PVR) is known to be elevated in patients with ARDS (Tables 2 and 3). Zapol and Jones were the first to document that raised pulmonary vascular resistance was a common finding in patients with severe respiratory failure [9]. They observed that pulmonary vascular resistance tended to fall in survivors but remained elevated in those who died. This is the only study to report pulmonary haemodynamic indices longitudinally.

**Table 3:** Studies of indices of RVD and outcome in ARDS

**Study period**	**Recruitment period**	**Number**	**Data source**	**RVD definition**	**Independent predictor of mortality?**
Jardin and Vieillard-Baron [17]	1980 to 2006	352	Echo	RV:LV EDA >0.6 and IVS flattening at end-systole	N
Monchi et al. [18]	1992 to 1995	259	PAC	RAP > PAOP	Y
Vieillard-Baron et al. [19]	1996 to 2001	75	Echo	RV:LV EDA >0.6 and IVS flattening at end-systole	N
Osman et al. [13]	1999 to 2001	145	PAC	CVP > PAOP and MPAP > 25 mmHg and SVI < 30 ml m^−2^	N
Bull et al. [5]	2000 to 2005	501	PAC	CVP > PAOP	N
Boissier et al. [20]	2004 to 2009	226	Echo	RV:LV EDA >0.6 and IVS flattening at end-systole	Y
Lhéritier et al. [21]	2009 to 2012	200	Echo	RV:LV EDA >0.6 and IVS flattening at end-systole	N

CVP, central venous pressure; EDA, end-diastolic area; IVS, interventricular septum; MPAP, mean pulmonary artery pressure; PAOP, pulmonary artery occlusion pressure; RAP, right atrial pressure; SVI, stroke volume index.

Zapol and Jones subsequently documented a three-fold elevation in PVR in patients with ARDS [22]. These findings were replicated by Villar et al. They showed a marked elevation in pulmonary vascular resistance in association with reduced right ventricular cardiac index (CI) in 30 patients with ARDS [10].

In a secondary analysis of the haemodynamic data from the fluid and catheter treatment (FACTT) trial of 501 patients with ARDS who were managed with a pulmonary artery catheter, Bull et al. showed that the transpulmonary gradient (mPAP-pulmonary arterial occlusion pressure (PAOP)) and the pulmonary vascular resistance index (mPAP-PAOP/CI) were the only pulmonary haemodynamic indices that showed a significant difference between those who died and those who survived. Multivariate analyses showed them to be independent predictors of mortality in ARDS [5]. They used the term ‘pulmonary vascular dysfunction’ to describe these two variables. Covariates in their multivariate analyses included sex, race, age, APACHE II score, the presence of shock at baseline, level of positive end-expiratory pressure (PEEP), the PaO_2_:FiO_2_ ratio and fluid treatment strategy. They did not find any difference in P:F ratios, PASP, PADP, mPAP, PAOP or cardiac index between those who survived with ARDS and those who did not. The Pplat and PEEP levels were not different among the groups. It is worth noting that 21% of the screened patients were excluded because they had a pulmonary artery catheter in place at the time of randomization and that 30% of the enrolled patients showed a PAOP > 18 mmHg at enrollment, therefore not meeting the ABC definition of ARDS. This may have explained why the PAP-PAOP gradient may have been significant, when PAP was not.

There are marked differences among these studies, with some showing that pulmonary arterial pressure is independently associated with mortality, and in others’ findings, it is not. Similarly, increased PVR was found to be a predictor of adverse outcome in some studies and not in others.

Before considering these discrepancies in more detail, it is helpful to examine the relationship between PAP and PVR in healthy subjects and to look at the pathophysiology of elevated pulmonary vascular resistance.

#### Physiology of pulmonary haemodynamics

There is a complex, non-linear relationship between pulmonary arterial pressure and pulmonary vascular resistance in normal, non-diseased lungs.

In the lungs, the PVR is conventionally calculated as follows:

(1) PVR=mPAP−LAP/CO

where PVR = pulmonary vascular resistance, mPAP = mean pulmonary arterial pressure, LAP = left atrial pressure and CO = cardiac output.

**Figure 1:**
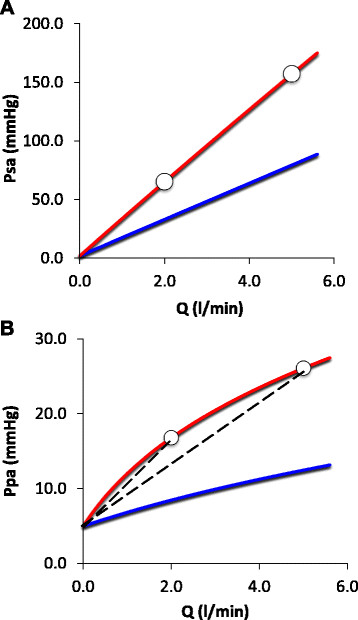
**Mean arterial pressure plotted against flow (cardiac output) in the systemic (A) and pulmonary (B) circulations.** The blue curve in each panel represents the normal condition of the circulation, and the red curve a hypertensive condition. **(A)** In the systemic circulation, the mean pressure (P)-flow (Q) plot is well described as a linear (Ohmic) relationship. The two points identified (open circles) show a normal cardiac output and a reduced cardiac output, respectively, in the hypertensive condition. At each of these cardiac outputs, it is clear that the ratio of P to Q is the same and therefore can be used to easily characterise the resistance of the systemic circulation. **(B)** In the pulmonary circulation, the plot of mean pressure against flow is curvilinear with an intercept on the pressure axis that is equal to left atrial pressure. The blue curve represents a normal pressure flow curve (healthy lung), while the red curve represents pressure flow curve in the presence of hypoxic pulmonary hypertension. The two points identified (open circles) show a normal cardiac output and a reduced cardiac output, respectively, in the hypertensive condition. At each cardiac output the pulmonary vascular resistance, (Ppa-LAP)/Q, is illustrated as the slope of the straight dashed line. Even though the two points are each on the same pressure flow curve, the calculated pulmonary vascular resistance is different at the different cardiac outputs. Psa, systemic arterial pressure (mean); Ppa, pulmonary arterial pressure (mean); Q, cardiac output (flow).

In the systemic circulation, an Ohmic relationship between driving pressure and flow through the blood vessel provides a reasonable approximation (Figure 1A). In such a system, the plot of pressure against flow is a straight line passing through the origin and the resistance to flow is well characterised as the ratio of the arterial pressure to the flow (cardiac output) at all points along the pressure flow line.

In contrast, the blood flow through the lungs is not well described by a linear relationship passing through the origin but by a curvilinear plot that has a positive intercept on the pressure axis (Figure 1B). This curvilinear relationship arises because of the marked distensibility of the pulmonary vasculature. An increase in pulmonary arterial pressure results in an increased flow due both to the higher driving pressure and the distension of the vessels so that the diameter of the vascular lumen is increased. Thus, increases in pulmonary arterial pressure have a disproportionate effect on pulmonary blood flow.

As a consequence, a reduction in cardiac output leads to an increase in the ratio of the pressure drop across the pulmonary circulation (PAP-LAP) to flow, even though there is no change in vasomotor tone (Figure 1B).

Blood flow through the lungs also depends on the transmural pressure in the pulmonary vessels (pressure within lumen minus airway pressure) to a much greater extent than in systemic vessels. *Airway pressure* can have a marked effect on pulmonary blood flow, as originally determined by West [23].

*Lung volume* has an important effect on PVR which is independent of vascular transmural pressure. Whittenberger et al. [24] described how low (near residual volume) lung volumes were associated with a slight elevation in PVR (extra-alveolar vessels are narrowed) and high lung volumes (near total lung capacity) were associated with the highest PVR (alveolar capillaries are stretched). This contributes to a marked elevation in pulmonary vascular resistance, even if the vascular transmural pressure is kept constant [25]. Pulmonary arterial pressure is not only affected by changes in pulmonary vascular resistance but also changes in right ventricular (RV) output. RV output, in turn, is affected by factors that are extrinsic to the lung.

It is evident, even from this brief summary, that pulmonary arterial pressure and pulmonary vascular resistance cannot be used as interchangeable measures of the state of pulmonary haemodynamics in patients with ARDS. For a comprehensive review of this problem of interpreting changes in pulmonary vascular resistance, the reader is referred to the work of Vesprille and Naeije [26],[27].

#### Mechanisms of increased PVR in ARDS

Many of the candidate mechanisms that explain an elevation in PVR in ARDS have been recently reviewed [28]. We will highlight the pathophysiology of some of these mechanisms.

### Lung disease-related mechanisms

#### HPV

Bradford and Dean were among the first to recognise that hypoxia resulted in sustained elevations in pulmonary arterial pressure [29]. The mechanisms that underlie hypoxic pulmonary vasoconstriction (HPV) are complex and primarily relate to intracellular increases in calcium concentration and Rho kinase-mediated sensitisation in pulmonary arterial smooth muscle cells [30]–[33].

HPV causes an increase in PVR to 100% to 150% of baseline when healthy volunteers are exposed to hypoxia (PO_2_ 50 mmHg) [34]. Marshall et al. have shown that when HPV is acutely reduced in ARDS by the administration of 100% inspired oxygen, pulmonary arterial pressure was reduced by the order of 10% to 15% from its peak [35]. This may be an underestimate of the extent of HPV in the lung, as it does not take into account the contribution of HPV in non-ventilated lung units.

To assess the contribution of non-ventilated lung units to HPV, Benzing et al. took a group of 11 patients with severe ARDS treated by veno-venous extracorporeal lung assist and ventilated them with an FiO_2_ of 1.0 for a period of 20 min prior to taking measurements (thereby minimising HPV in ventilated lung units).

They then manipulated the mixed venous partial pressure of oxygen (PvO_2_) by adjusting the proportion of blood flow diverted through the oxygenator in order to assess HPV in non-ventilated regions. When PvO_2_ was high (83.6 ± 2.4 mmHg), the total lung PVR was 339 (±29) dyne.s.cm^−5^.m^2^ and increased by 28.9% to 437 (±36) dyne.s.cm^−5^.m^2^ when PvO_2_ was reduced to low values (46.6 ± 0.1 mmHg) [36], clearly demonstrating that HPV in non-ventilated lung units contributes significantly to the increase in pulmonary vascular resistance in ARDS.

In addition to the influence of HPV, disruption of the endothelium in ARDS results in an alteration in the normal balance of mediators of vasodilation (NO, prostacyclin) and vasoconstriction (thromboxane, leukotrienes, endothelin, serotonin, angiotensin II) favouring vasoconstriction. These factors have been reviewed recently [28],[37].

#### Thrombosis

Tomashefski et al., in a landmark post-mortem study of 22 patients with ARDS, found that 19 patients had evidence of microthrombi. Nineteen had macrothrombi in the pulmonary arterial and capillary vessels [38]. They also found endothelial injury in all stages of ARDS in all cases on both standard histological preparations and electron microscopy. There is now ample evidence supporting the concept of lung injury causing local, as opposed to systemic, coagulation in ARDS [28],[39],[40]. Tissue factor (TF) is released from endothelial cells that have been injured, in response to a variety of pro-inflammatory stimuli [41]. TF is a strong activator of the extrinsic clotting cascade. Increased activation of pro-coagulant processes occurs in the lung in ARDS and does not result from the systemic activation of coagulation (such as is seen in sepsis) [42],[43]. Animal data suggest that blockade of the TF-factor VIIa-factor Xa complex may reduce the degree of pulmonary hypertension in ARDS [44]. Levels of protein C, a natural anti-coagulant, are also reduced in ARDS [45] while levels of plasminogen activator inhibitor-1 are increased in ARDS patients, and both are prognostic of increased mortality in ARDS [46]. More recently, biomarkers of coagulation and inflammation have been shown to provide good discrimination for the diagnosis of patients with ARDS [47],[48], and analysis of SARS-CoV infection in laboratory models has shown that the delicate balance between coagulation and fibrinolysis is shifted towards fibrin deposition during infection leading to ARDS [49]. Therapies targeting this pulmonary coagulopathy may also have an anti-inflammatory effect and attenuate the severity of ARDS [50].

Therefore, ARDS represents a procoagulant, anti-fibrinolytic phenotype and results in the local formation of microthrombi, which may, in turn, act to increase the pulmonary vascular resistance by the mechanical obstruction of blood flow.

#### Vascular remodelling

Fibroproliferation is a characteristic of the late stage of ARDS, and is present in approximately 55% of patients who die of this condition [51]. It is associated with increased mortality, and the presence of fibrosis on thin cut CT scan has been used to predict outcome in ARDS [52],[53]. In a small post-mortem study of the lungs of patients who had died with ‘severe respiratory failure’, Zapol et al. demonstrated that there is increasing destruction of the capillary bed as ARDS progresses, which may contribute to elevations in the PVR of the same patients measured ante-mortem [54]. Many mediators have been linked to the fibroproliferative response, but those that have an association with vascular effects include angiotensin II and vascular endothelial growth factor (VEGF) [55]–[57].

Tomashefski et al. [38] noted that there was electron microscopy evidence for extensive vascular remodelling in ARDS. The intermediate phase was characterised by fibrocellular obliteration of the arteries, veins and even lymphatic vessels. In the late stage, vascular remodelling was associated with distorted, tortuous arteries and veins. These tortuous channels were concentrated in regions of dense or irregular fibrosis. The number of capillaries was reduced, and they were often dilated. Muscularisation of the arteries was identified in the intermediate phase and was very marked in the late phase. This mechanical disruption of the course of blood vessels is likely to contribute to the sustained elevation in PVR seen in non-survivors.

### Ventilator-related mechanism of raised PVR in ARDS

#### PEEP

A key difference between normal lungs and injured lungs in ARDS is the use of mechanical ventilation in the latter, requiring the application of PEEP and positive inspiratory plateau pressures. When PEEP is applied to a diseased lung, the change in PVR is determined by the balance between overdistension of lung units and recruitment of areas with previously low numbers of open alveoli. When the number of open alveoli increases following a recruitment manoeuvre and application of high PEEP, then PVR may even fall in keeping with Whittenberger’s-U shaped relationship between pulmonary vascular resistance and lung volume. Any increase in ventilated alveolar area may also reduce HPV. Canada et al., found that the pulmonary vascular resistance index (PVRi) was lowest at 5 cm H_2_O in the normal lung but 10 cm H_2_O had to be applied to the injured lung in order to achieve minimal PVRi [58]. Above ‘optimal PEEP’ levels, the PVR increased, presumably due to compression of intra-alveolar capillaries by the increased airway pressure resulting in an increase in zone 1 and 2 characteristics [23],[59].

#### Plateau pressure

There are very few studies which have measured pulmonary vascular resistance in ARDS patients ventilated with lower tidal volumes, perhaps due to the reduction in the use of the pulmonary artery catheter just as lung-protective ventilation was gaining widespread acceptance [60].

Limitation of plateau pressures has, however, been shown to be associated with lower rates of right ventricular failure than in historical studies [17],[19]. The application of higher tidal volume to the patients in these studies was associated with a significant increase in right ventricular afterload [61].

#### Is PVR affected by the way patients are ventilated?

There is currently no evidence to suggest that one mode of ventilation has more or less effect than any other mode on pulmonary vascular haemodynamics. Any effect of the mode of ventilation on PVR is likely to be related to the amount of PEEP and plateau pressure that is applied.

### Why do the studies of pulmonary haemodynamics report inconsistent relationships with mortality?

As is apparent, PVR is directly influenced by factors that are intrinsic to the lung and can be increased by the pathophysiological insults that occur in ARDS. In contrast, PAP is affected both by factors extrinsic to the lung (e.g. RV output preload and contractility) and by factors intrinsic to the lung (PVR).

In clinical practice, there is considerable variability in the preload of patients with ARDS. Both volume loading and venous tone have a considerable influence on the amount of venous return reaching the heart. The presence of sepsis and the use of vasopressors will both affect venous tone. Likewise, raised intra-thoracic pressure can have a compressive effect on the intra-thoracic veins, including the superior and inferior venae cavae [62] and limit venous return in patients with ARDS. Sepsis-induced cardiac dysfunction may result in RV impairment in as many as 24% of patients [63].

The studies in Table 2 have reached different conclusions about the significance of PAP and PVR and their relationship to outcome in ARDS. What might account for these differences?

All except one of the studies quoted are observational in nature and did not employ standard patient management protocols. The studies were not designed to answer specific questions about the nature of pulmonary haemodynamics in ARDS, and the data were drawn from patients who were managed differently in terms of mechanical ventilation (mode and pressures applied), fluid status and vasopressor use, all of which adds to the statistical noise when trying to draw useful conclusions.

Bull et al.’s data came from patients who all had a standardised approach to ventilator management (in particular the use of low tidal volume ventilation), pulmonary artery catheter data acquisition as well as fluid management. Bull et al.’s study, the largest in the modern era of ‘protective ventilation’ found no association between PAP and outcome but showed a highly significant and independent link between two indices of pulmonary vascular dysfunction (mPAP-PAOP and PVRi) and mortality.

PVR is primarily affected by factors that are intrinsic to the lung, while PAP is influenced by both PVR and RV preload and contractility. When the variability in management was controlled for (as in Bull et al.’s study), the measured PVR was more likely to have reflected the vascular changes induced by the disease process in ARDS. This is because the protocol standardised many of the extrinsic factors (airway pressure, tidal volume, fluid loading) that can influence PA pressure independently of changes in pulmonary vascular resistance. Importantly, in this well-controlled study, indices of elevated pulmonary vascular resistance were found to independently predict greater mortality in ARDS.

### PVR, ARDS and mortality - association or causation?

This highly significant association between mortality and measures of pulmonary vascular resistance, in a carefully controlled study, raises the question as to whether PVD directly causes increased mortality or is it associated with mortality.

There are two potential mechanisms by which an elevation in PVR could cause mortality in ARDS. Either it results in right ventricular failure, with subsequent multi-organ dysfunction or it exacerbates the acute lung injury directly.

### Is RVD the cause of increased mortality in ARDS?

The right ventricle is more sensitive to acute increases in its afterload than the left ventricle. We know from studies of major pulmonary embolism, that a *normal* right ventricle cannot acutely generate pulmonary pressures greater than 40 mmHg (mean) and quickly fails in this clinical context [64]. Is the same true for patients with ARDS?

Sustained pulmonary hypertension may result in right ventricular failure (RVF) in ARDS patients [65]. Over the years, the incidence of right ventricular dysfunction (RVD) has declined as improvements in mechanical ventilation have been adopted and lessened the intrathoracic airway pressure in patients with ARDS [17],[19], but RVD is variably defined and diagnosed among studies which makes comparison difficult.

Clinically, right ventricular failure has no agreed definition, but criteria (using pulmonary artery catheter data) include pulmonary hypertension associated with an RV cardiac index <2.5 L min^−1^ m^−2^ and a right atrial pressure >8 mmHg [9]. Using these criteria, Osman et al. found an incidence of right ventricular failure of 9.6% in 145 patients with ARDS [13]. The presence of RVF was not associated with death. In Bull et al.’s analysis of 501 patients with ARDS, they reported an incidence of right ventricular failure (RVF) of 12% (using Monchi’s definition of right atrial pressure > pulmonary artery occlusion pressure [5],[18]); RVF was not predictive of mortality.

The presence of RVF can also be inferred using echocardiographic criteria. Acute cor pulmonale (ACP) has been defined as the presence of RV dilation (ratio of RV end-diastolic area to left ventricle end-diastolic area >0.6) in association with dyskinesia of the interventicular septum in response to an increased afterload [19]. Jardin et al. originally described the two-dimensional echo characteristics in a group of 23 patients with acute respiratory failure, showing that the right ventricular end-diastolic area increased as the PVRi (measured using a PAC) increased and RV stroke volume declined [66].

Vieillard-Baron et al. have demonstrated an incidence of echocardiographic cor pulmonale of 25% in a study of 75 patients with ARDS [19],[61]. However, ACP was found to be reversible in those patients whose ARDS resolved, and it did not have a negative prognostic significance. Similar results were found by Cepkova in a study of 42 patients with acute lung injury [67].

In a retrospective analysis of 352 patients with ARDS admitted to their unit since 1980, Jardin’s group found a correlation between increasing levels of plateau pressure and the incidence of acute cor pulmonale [17]. As measured plateau pressure increased, the incidence of ACP rose up to 42% with plateau pressures of >35 cm H_2_O. While they also noted an association between the presence of ACP and mortality in the overall group, this did not hold true when the airway pressure was aggressively limited, in line with current practice [19].

Vieillard-Baron’s group [17],[19],[61],[68],[69] have suggested that the increases in RV afterload due to elevations in PEEP and plateau pressure, as well the underlying lung injury, result in RV dysfunction that is sufficient to increase mortality. This reflects what we know of the pathophysiology of pulmonary embolism, but the evidence is not as definitive in ARDS. The presence of ACP has not been consistently demonstrated to be associated with excess mortality in ARDS in the modern era of protective ventilation. Perhaps this is because the authors modified their approach to mechanical ventilation in these studies when ACP was recognised, in order to limit the distension of the right ventricle by reducing the airway pressures (PEEP and plateau) and putting the patient in a prone position [17],[21]. Recent echocardiographic derived data on right ventricular dysfunction from Boissier et al. [20] suggest that even when tidal volume and plateau pressure are limited in line with best practice, the incidence of ACP in ARDS is still 22% and is independently associated with mortality in spite of greater use of prone positioning and nitric oxide. Lheritier et al. [21] found a similar incidence of ACP (22.5%) in moderate to severe ARDS patients ventilated with a lung protective strategy, *but* they could not find an association between the presence of ACP and outcome. In both studies, the groups with ACP had a higher use of nitric oxide and prone positioning compared to those without ACP. It is unclear what accounts for the different findings in these studies.

The relationship between ACP/RVD and outcome in ARDS is therefore unclear, and it remains to be determined.

### Is pulmonary vascular dysfunction a cause of ARDS?

It is worth asking the question as to whether there is a plausible mechanistic basis that would allow pulmonary vascular dysfunction to worsen ARDS. High-altitude pulmonary oedema (HAPE) is a condition that occurs in previously healthy individuals within 2 to 4 days after rapid ascent above altitudes of 3,500 to 4,000 m [70],[71]. While it is not a form of ARDS, it is a severe form of non-cardiogenic pulmonary oedema, which can develop in susceptible individuals (5% to 10% of the normal population) in the presence of hypoxia alone [72].

Individuals who develop HAPE have an increased degree of HPV compared to unaffected members of the population. Pulmonary artery pressure at an altitude of 4,559 m is about 30% to 50% higher in individuals who are prone to HAPE compared with non-susceptible controls, and this higher pressure precedes oedema formation [70]. The increase in HPV can also be demonstrated at low altitude in susceptible individuals exposed to a brief hypoxic challenge [73],[74].

Lowering pulmonary artery pressure during the ascent to high altitude can prevent HAPE. A non-specific pulmonary vasodilator (nifedipine) [70] or the phosphodiesterase-5-inhibitor tadalafil [75] reduced the prevalence of pulmonary oedema in HAPE-susceptible individuals after rapid ascent to 4,559 m from 60% to about 10%. This suggests that excessive HPV may contribute to the development of acute oedema, possibly by redistributing pulmonary blood flow away from areas with high degrees of HPV to other sections of the lung, with resultant hyper-perfusion, endothelial injury and capillary leak. This causes a secondary inflammation which is clinically indistinguishable from ARDS [76].

The finding that a subset of the population is prone to the development of non-cardiogenic pulmonary oedema, as a result of exposure to hypoxia alone, is of relevance to our understanding of ARDS. ARDS is characterised by heterogeneous areas of alveolar hypoxia and inappropriate vascular responses to these areas of hypoxia may partially explain the finding that individuals with pulmonary vascular dysfunction have worse outcomes in ARDS. There is, as of yet, no evidence to support this hypothesis in the general population who present with ARDS.

### Is PVD a marker of the severity of ARDS?

As patients recover from ARDS, there is resolution of the pulmonary vascular dysfunction. Many of the mechanisms of PVD in ARDS (the release of multiple vasoactive mediators, vascular remodelling and the formation of vaso-occlusive microthrombi) are caused by the disruption of the normal endothelial-inflammation-coagulation pathways. PVD may be a good summative index of vascular damage from these mechanisms. Nuckton et al. has previously reported that an increased dead space fraction was associated with increased mortality in ARDS [77], which they postulated might be due to injury to the pulmonary capillaries from inflammation and thrombosis and obstruction of pulmonary blood flow in the extra-alveolar pulmonary circulation. There is evidence that extra-pulmonary organ dysfunction in ARDS is caused by the systemic inflammatory response, which in turn is driven by the initiating pulmonary injury [78]. If PVD is primarily a downstream result of the activation of the inflammatory-coagulation cascade in the lung, then, the reason it is associated with mortality in ARDS may be because it reflects the severity of the underlying inflammatory process. This hypothesis may also help to explain why PVD is associated with mortality in well-controlled studies of patients with ARDS whereas right ventricular dysfunction has not been consistently shown to be associated with mortality.

ARDS studies are rarely adequately powered to look at mortality as they do not recruit sufficient numbers of patients to be able to draw valid conclusions. Using PVD as an index of disease severity might allow researchers an additional way to stratify the severity of lung injury and to test the efficacy of new treatments for ARDS by measuring the change in PVD, which is known to improve as the patient recovers from lung injury. In order to develop new treatments for ARDS, we need better methods for examining their efficacy. Using PVD as an endpoint might improve the predictive value of phase II trials prior to embarking on full scale clinical studies of new treatments. Assessment of pulmonary vascular resistance may be possible using non-invasive echocardiographic technology [79] which would increase the applicability of this approach and may be worth pursuing.

## Conclusions

Pulmonary vascular dysfunction is an independent predictor of mortality in ARDS. An examination of the physiology of pulmonary haemodynamics in ARDS helps to explain why it may be a clearer mortality signal, when compared to the inconsistent link between mortality and pulmonary arterial pressure or right ventricular dysfunction.

Further study is needed to determine precisely the dominant pathways involved in causing PVD in ARDS. This is an area of research that may yet lead to greater understanding of the complex interplay between the pulmonary circulation, endothelial dysfunction and activation of the inflammatory-coagulation cascades that underlie ARDS.

## Abbreviations

ACP: acute cor pulmonale

ARDS: acute respiratory distress syndrome

CO: cardiac output

HPV: hypoxic pulmonary vasoconstriction

LAP: left atrial pressure

mPAP: mean pulmonary arterial pressure

NO: nitric oxide

P:F: ratio of partial pressure of oxygen to fraction of inspired oxygen

Pplat: plateau pressure

PAC: pulmonary artery catheter

PADP: pulmonary arterial diastolic pressure

PAOP: pulmonary arterial occlusion pressure

PAP: pulmonary arterial pressure

PASP: pulmonary arterial systolic pressure

PEEP: positive end-expiratory pressure

PVD: pulmonary vascular dysfunction

PVR: pulmonary vascular resistance

RV: right ventricle

RVD: right ventricular dysfunction

RVF: right ventricular failure

TPG: transpulmonary gradient

## Competing interests

The authors declare that they have no competing interests.

## Authors’ contributions

DR was responsible for writing, editing and reviewing the majority of the manuscript. SF wrote and reviewed the section on high-altitude pulmonary oedema. PMcL was responsible for the concept for the review, editing and final review of the manuscript. All authors read and approved the final manuscript.

## References

[B1] Ware LB Matthay MA The acute respiratory distress syndrome N Engl J Med 2000 342 1334 1349 10793167 10.1056/NEJM200005043421806

[B2] Ventilation with lower tidal volumes as compared with traditional tidal volumes for acute lung injury and the acute respiratory distress syndrome N Engl J Med 2000 342 1301 1308 10793162 10.1056/NEJM200005043421801

[B3] Rubenfeld GD Caldwell E Peabody E Weaver J Martin DP Neff M Stern EJ Hudson LD Incidence and outcomes of acute lung injury N Engl J Med 2005 353 1685 1693 16236739 10.1056/NEJMoa050333

[B4] Phua J Badia JR Adhikari NK Friedrich JO Fowler RA Singh JM Scales DC Stather DR Li A Jones A Gattas DJ Hallett D Tomlinson G Stewart TE Ferguson ND Has mortality from acute respiratory distress syndrome decreased over time? A systematic review Am J Respir Crit Care Med 2009 179 220 227 19011152 10.1164/rccm.200805-722OC

[B5] Bull TM Clark B McFann K Moss M Pulmonary vascular dysfunction is associated with poor outcomes in patients with acute lung injury Am J Respir Crit Care Med 2010 182 1123 1128 20558628 10.1164/rccm.201002-0250OC PMC3001255

[B6] Squara P Dhainaut JF Artigas A Carlet J Hemodynamic profile in severe ARDS: results of the European Collaborative ARDS Study Intensive Care Med 1998 24 1018 1028 9840234 10.1007/s001340050710

[B7] Galie N Hoeper MM Humbert M Torbicki A Vachiery JL Barbera JA Beghetti M Corris P Gaine S Gibbs JS Gomew-Sanchez MA Jondeau G Klepetko W Opitz C Peacock A Rubin L Zellweger M Simmoneau G Guidelines for the diagnosis and treatment of pulmonary hypertension: the Task Force for the Diagnosis and Treatment of Pulmonary Hypertension of the European Society of Cardiology (ESC) and the European Respiratory Society (ERS), endorsed by the International Society of Heart and Lung Transplantation (ISHLT) Eur Heart J 2009 30 2493 2537 19713419 10.1093/eurheartj/ehp297

[B8] Cepkova M Kapur V Ren X Quinn T Zhuo H Foster E Liu KD Matthay MA Pulmonary dead space fraction and pulmonary artery systolic pressure as early predictors of clinical outcome in acute lung injury Chest 2007 132 836 842 17573490 10.1378/chest.07-0409

[B9] Zapol WM Snider MT Pulmonary hypertension in severe acute respiratory failure N Engl J Med 1977 296 476 480 834225 10.1056/NEJM197703032960903

[B10] Villar J Blazquez MA Lubillo S Quintana J Manzano JL Pulmonary hypertension in acute respiratory failure Crit Care Med 1989 17 523 526 2721210 10.1097/00003246-198906000-00007

[B11] Suchyta MR Clemmer TP Elliott CG Orme JF Jr Weaver LK The adult respiratory distress syndrome. A report of survival and modifying factors Chest 1992 101 1074 1079 1555423 10.1378/chest.101.4.1074

[B12] Hemmila MR Rowe SA Boules TN Miskulin J McGillicuddy JW Schuerer DJ Haft JW Swaniker F Arbabi S Hirschl RB Bartlett RH Extracorporeal life support for severe acute respiratory distress syndrome in adults Ann Surg 2004 240 595 605 discussion 605–597 15383787 10.1097/01.sla.0000141159.90676.2d PMC1356461

[B13] Osman D Monnet X Castelain V Anguel N Warszawski J Teboul JL Richard C Incidence and prognostic value of right ventricular failure in acute respiratory distress syndrome Intensive Care Med 2009 35 69 76 18839137 10.1007/s00134-008-1307-1

[B14] Beiderlinden M Kuehl H Boes T Peters J Prevalence of pulmonary hypertension associated with severe acute respiratory distress syndrome: predictive value of computed tomography Intensive Care Med 2006 32 852 857 16614811 10.1007/s00134-006-0122-9

[B15] Badesch DB Champion HC Sanchez MA Hoeper MM Loyd JE Manes A McGoon M Naeije R Olschewski H Oudiz RJ Torbicki A Diagnosis and assessment of pulmonary arterial hypertension J Am Coll Cardiol 2009 54 S55 S66 19555859 10.1016/j.jacc.2009.04.011

[B16] McNeil K Dunning J Morrell NW The pulmonary physician in critical care. 13: the pulmonary circulation and right ventricular failure in the ITU Thorax 2003 58 157 162 12554902 10.1136/thorax.58.2.157 PMC1746562

[B17] Jardin F Vieillard-Baron A Is there a safe plateau pressure in ARDS? The right heart only knows Intensive Care Med 2007 33 444 447 17268795 10.1007/s00134-007-0552-z

[B18] Monchi M Bellenfant F Cariou A Joly LM Thebert D Laurent I Dhainaut JF Brunet F Early predictive factors of survival in the acute respiratory distress syndrome. A multivariate analysis Am J Respir Crit Care Med 1998 158 1076 1081 9769263 10.1164/ajrccm.158.4.9802009

[B19] Vieillard-Baron A Schmitt JM Augarde R Fellahi JL Prin S Page B Beauchet A Jardin F Acute cor pulmonale in acute respiratory distress syndrome submitted to protective ventilation: incidence, clinical implications, and prognosis Crit Care Med 2001 29 1551 1555 11505125 10.1097/00003246-200108000-00009

[B20] Boissier F Katsahian S Razazi K Thille AW Roche-Campo F Leon R Vivier E Brochard L Vieillard-Baron A Brun-Buisson C Mekontso Dessap A Prevalence and prognosis of cor pulmonale during protective ventilation for acute respiratory distress syndrome Intensive Care Med 2013 39 1725 1733 23673401 10.1007/s00134-013-2941-9

[B21] Lheritier G Legras A Caille A Lherm T Mathonnet A Frat JP Courte A Martin-Lefevre L Gouello JP Amiel JB Garot D Vignon P Prevalence and prognostic value of acute cor pulmonale and patent foramen ovale in ventilated patients with early acute respiratory distress syndrome: a multicenter study Intensive Care Med 2013 39 1734 1742 23860806 10.1007/s00134-013-3017-6

[B22] Zapol WM Jones R Vascular components of ARDS. Clinical pulmonary hemodynamics and morphology Am Rev Respir Dis 1987 136 471 474 3619211 10.1164/ajrccm/136.2.471

[B23] West JB Regional differences in the lung Chest 1978 74 426 437 699656 10.1378/chest.74.4.426

[B24] Whittenberger JL Mc GM Berglund E Borst HG Influence of state of inflation of the lung on pulmonary vascular resistance J Appl Physiol 1960 15 878 882 13784949 10.1152/jappl.1960.15.5.878

[B25] Hakim TS Michel RP Chang HK Effect of lung inflation on pulmonary vascular resistance by arterial and venous occlusion J Appl Physiol 1982 53 1110 1115 6757207 10.1152/jappl.1982.53.5.1110

[B26] Versprille A Pulmonary vascular resistance. A meaningless variable Intensive Care Med 1984 10 51 53 6715677 10.1007/BF00297557

[B27] Naeije R Pulmonary vascular resistance. A meaningless variable? Intensive Care Med 2003 29 526 529 12800831 10.1007/s00134-003-1693-3

[B28] Price LC McAuley DF Marino PS Finney SJ Griffiths MJ Wort SJ Pathophysiology of pulmonary hypertension in acute lung injury Am J Physiol Lung Cell Mol Physiol 2012 302 L803 L815 22246001 10.1152/ajplung.00355.2011 PMC3362157

[B29] Bradford JR Dean HP The pulmonary circulation J Physiol 1894 16 34 158 125 16992161 10.1113/jphysiol.1894.sp000493 PMC1514499

[B30] Moudgil R Michelakis ED Archer SL Hypoxic pulmonary vasoconstriction J Appl Physiol 2005 98 390 403 15591309 10.1152/japplphysiol.00733.2004

[B31] Mark Evans A Ward JP Hypoxic pulmonary vasoconstriction–invited article Adv Exp Med Biol 2009 648 351 360 19536499 10.1007/978-90-481-2259-2_40

[B32] Aaronson PI Robertson TP Knock GA Becker S Lewis TH Snetkov V Ward JP Hypoxic pulmonary vasoconstriction: mechanisms and controversies J Physiol 2006 570 53 58 16254010 10.1113/jphysiol.2005.098855 PMC1464287

[B33] Hyvelin JM Howell K Nichol A Costello CM Preston RJ McLoughlin P Inhibition of Rho-kinase attenuates hypoxia-induced angiogenesis in the pulmonary circulation Circ Res 2005 97 185 191 15961717 10.1161/01.RES.0000174287.17953.83

[B34] Dorrington KL Clar C Young JD Jonas M Tansley JG Robbins PA Time course of the human pulmonary vascular response to 8 hours of isocapnic hypoxia Am J Physiol 1997 273 H1126 H1134 9321798 10.1152/ajpheart.1997.273.3.H1126

[B35] Marshall BE Hanson CW Frasch F Marshall C Role of hypoxic pulmonary vasoconstriction in pulmonary gas exchange and blood flow distribution. 2. Pathophysiology Intensive Care Med 1994 20 379 389 7930036 10.1007/BF01720916

[B36] Benzing A Mols G Brieschal T Geiger K Hypoxic pulmonary vasoconstriction in nonventilated lung areas contributes to differences in hemodynamic and gas exchange responses to inhalation of nitric oxide Anesthesiology 1997 86 1254 1261 9197293 10.1097/00000542-199706000-00005

[B37] Moloney ED Evans TW Pathophysiology and pharmacological treatment of pulmonary hypertension in acute respiratory distress syndrome Eur Respir J 2003 21 720 727 12762363 10.1183/09031936.03.00120102

[B38] Tomashefski JF Jr Davies P Boggis C Greene R Zapol WM Reid LM The pulmonary vascular lesions of the adult respiratory distress syndrome Am J Pathol 1983 112 112 126 6859225 PMC1916312

[B39] Maniatis NA Kotanidou A Catravas JD Orfanos SE Endothelial pathomechanisms in acute lung injury Vascul Pharmacol 2008 49 119 133 18722553 10.1016/j.vph.2008.06.009 PMC7110599

[B40] Levi M ten Cate H van der Poll T Endothelium: interface between coagulation and inflammation Crit Care Med 2002 30 S220 S224 12004239 10.1097/00003246-200205001-00008

[B41] Scarpati EM Sadler JE Regulation of endothelial cell coagulant properties. Modulation of tissue factor, plasminogen activator inhibitors, and thrombomodulin by phorbol 12-myristate 13-acetate and tumor necrosis factor J Biol Chem 1989 264 20705 20713 2555368

[B42] Prabhakaran P Ware LB White KE Cross MT Matthay MA Olman MA Elevated levels of plasminogen activator inhibitor-1 in pulmonary edema fluid are associated with mortality in acute lung injury Am J Physiol Lung Cell Mol Physiol 2003 285 L20 L28 12730079 10.1152/ajplung.00312.2002

[B43] Bastarache JA Wang L Geiser T Wang Z Albertine KH Matthay MA Ware LB The alveolar epithelium can initiate the extrinsic coagulation cascade through expression of tissue factor Thorax 2007 62 608 616 17356058 10.1136/thx.2006.063305 PMC2117249

[B44] Welty-Wolf KE Carraway MS Miller DL Ortel TL Ezban M Ghio AJ Idell S Piantadosi CA Coagulation blockade prevents sepsis-induced respiratory and renal failure in baboons Am J Respir Crit Care Med 2001 164 1988 1996 11734456 10.1164/ajrccm.164.10.2105027

[B45] Ware LB Fang X Matthay MA Protein C and thrombomodulin in human acute lung injury Am J Physiol Lung Cell Mol Physiol 2003 285 L514 L521 12754194 10.1152/ajplung.00442.2002

[B46] Ware LB Matthay MA Parsons PE Thompson BT Januzzi JL Eisner MD Pathogenetic and prognostic significance of altered coagulation and fibrinolysis in acute lung injury/acute respiratory distress syndrome Crit Care Med 2007 35 1821 1828 17667242 10.1097/01.CCM.0000221922.08878.49 PMC2764536

[B47] Fremont RD Koyama T Calfee CS Wu W Dossett LA Bossert FR Mitchell D Wickersham N Bernard GR Matthay MA May AK Ware LB Acute lung injury in patients with traumatic injuries: utility of a panel of biomarkers for diagnosis and pathogenesis J Trauma 2010 68 1121 1127 20038857 10.1097/TA.0b013e3181c40728 PMC3347639

[B48] Ware LB Koyama T Zhao Z Janz DR Wickersham N Bernard GR May AK Calfee CS Matthay MA Biomarkers of lung epithelial injury and inflammation distinguish severe sepsis patients with acute respiratory distress syndrome Crit Care 2013 17 R253 24156650 10.1186/cc13080 PMC4056313

[B49] Gralinski LE Bankhead A 3rd Jeng S Menachery VD Proll S Belisle SE Matzke M Webb-Robertson BJ Luna ML Shukla AK Ferris MT Bolles M Chang J Aicher L Waters KM Smith RD Metz TO Law GL Katze MG McWeeney S Baric RS Mechanisms of severe acute respiratory syndrome coronavirus-induced acute lung injury MBio 2013 4 4 e00271 13 23919993 10.1128/mBio.00271-13 PMC3747576

[B50] Glas GJ Van Der Sluijs KF Schultz MJ Hofstra JJ Van Der Poll T Levi M Bronchoalveolar hemostasis in lung injury and acute respiratory distress syndrome J Thromb Haemost 2013 11 17 25 23114008 10.1111/jth.12047

[B51] Dos Santos CC Advances in mechanisms of repair and remodelling in acute lung injury Intensive Care Med 2008 34 619 630 18264692 10.1007/s00134-007-0963-x

[B52] Martin C Papazian L Payan MJ Saux P Gouin F Pulmonary fibrosis correlates with outcome in adult respiratory distress syndrome. A study in mechanically ventilated patients Chest 1995 107 196 200 7813276 10.1378/chest.107.1.196

[B53] Ichikado K Suga M Muranaka H Gushima Y Miyakawa H Tsubamoto M Johkoh T Hirata N Yoshinaga T Kinoshita Y Yamashita Y Sasaki Y Prediction of prognosis for acute respiratory distress syndrome with thin-section CT: validation in 44 cases Radiology 2006 238 321 329 16293804 10.1148/radiol.2373041515

[B54] Zapol WM Kobayashi K Snider MT Greene R Laver MB Vascular obstruction causes pulmonary hypertension in severe acute respiratory failure Chest 1977 71 306 307 836381 10.1378/chest.71.2_supplement.306

[B55] Medford AR Millar AB Vascular endothelial growth factor (VEGF) in acute lung injury (ALI) and acute respiratory distress syndrome (ARDS): paradox or paradigm? Thorax 2006 61 621 626 16807391 10.1136/thx.2005.040204 PMC1828639

[B56] Yang R Thomas GR Bunting S Ko A Ferrara N Keyt B Ross J Jin H Effects of vascular endothelial growth factor on hemodynamics and cardiac performance J Cardiovasc Pharmacol 1996 27 838 844 8761851 10.1097/00005344-199606000-00011

[B57] Azamfirei L Gurzu S Solomon R Copotoiu R Copotoiu S Jung I Tilinca M Branzaniuc K Corneci D Szederjesi J Kovacs J Vascular endothelial growth factor: a possible mediator of endothelial activation in acute respiratory distress syndrome Minerva Anestesiol 2010 76 609 616 20661201

[B58] Canada E Benumof JL Tousdale FR Pulmonary vascular resistance correlates in intact normal and abnormal canine lungs Crit Care Med 1982 10 719 723 6754259 10.1097/00003246-198211000-00004

[B59] Walther SM Domino KB Glenny RW Hlastala MP Positive end-expiratory pressure redistributes perfusion to dependent lung regions in supine but not in prone lambs Crit Care Med 1999 27 37 45 9934891 10.1097/00003246-199901000-00024

[B60] Koo KK Sun JC Zhou Q Guyatt G Cook DJ Walter SD Meade MO Pulmonary artery catheters: evolving rates and reasons for use Crit Care Med 2011 39 1613 1618 21494107 10.1097/CCM.0b013e318218a045

[B61] Vieillard-Baron A Jardin F Why protect the right ventricle in patients with acute respiratory distress syndrome? Curr Opin Crit Care 2003 9 15 21 12548024 10.1097/00075198-200302000-00004

[B62] Funk DJ Jacobsohn E Kumar A Role of the venous return in critical illness and shock: part II-shock and mechanical ventilation Crit Care Med 2013 41 573 579 23263572 10.1097/CCM.0b013e31827bfc25

[B63] Vieillard-Baron A Caille V Charron C Belliard G Page B Jardin F Actual incidence of global left ventricular hypokinesia in adult septic shock Crit Care Med 2008 36 1701 1706 18496368 10.1097/CCM.0b013e318174db05

[B64] Wood KE Major pulmonary embolism Crit Care Clin 2011 27 885 906 vi-vii 22082519 10.1016/j.ccc.2011.09.002

[B65] Greyson CR Pathophysiology of right ventricular failure Crit Care Med 2008 36 S57 S65 18158479 10.1097/01.CCM.0000296265.52518.70

[B66] Jardin F Gueret P Dubourg O Farcot JC Margairaz A Bourdarias JP Two-dimensional echocardiographic evaluation of right ventricular size and contractility in acute respiratory failure Crit Care Med 1985 13 952 956 2932300 10.1097/00003246-198511000-00035

[B67] Cepkova M Kapur V Ren X Quinn T Zhuo H Foster E Matthay MA Liu KD Clinical significance of elevated B-type natriuretic peptide in patients with acute lung injury with or without right ventricular dilatation: an observational cohort study Ann Intensive Care 2011 1 18 21906356 10.1186/2110-5820-1-18 PMC3224453

[B68] Mekontso Dessap A Charron C Devaquet J Aboab J Jardin F Brochard L Vieillard-Baron A Impact of acute hypercapnia and augmented positive end-expiratory pressure on right ventricle function in severe acute respiratory distress syndrome Intensive Care Med 2009 35 1850 1858 19652953 10.1007/s00134-009-1569-2 PMC3206087

[B69] Vieillard-Baron A Charron C Caille V Belliard G Page B Jardin F Prone positioning unloads the right ventricle in severe ARDS Chest 2007 132 1440 1446 17925425 10.1378/chest.07-1013

[B70] Bartsch P Maggiorini M Ritter M Noti C Vock P Oelz O Prevention of high-altitude pulmonary edema by nifedipine N Engl J Med 1991 325 1284 1289 1922223 10.1056/NEJM199110313251805

[B71] Hackett PH Roach RC High-altitude illness N Engl J Med 2001 345 107 114 11450659 10.1056/NEJM200107123450206

[B72] West JB The physiologic basis of high-altitude diseases Ann Intern Med 2004 141 789 800 15545679 10.7326/0003-4819-141-10-200411160-00010

[B73] Hultgren HN Grover RF Hartley LH Abnormal circulatory responses to high altitude in subjects with a previous history of high-altitude pulmonary edema Circulation 1971 44 759 770 5115068 10.1161/01.cir.44.5.759

[B74] Viswanathan R Jain SK Subramanian S Subramanian TA Dua GL Giri J Pulmonary edema of high altitude. II. Clinical, aerohemodynamic, and biochemical studies in a group with history of pulmonary edema of high altitude Am Rev Respir Dis 1969 100 334 341 5822044 10.1164/arrd.1969.100.3.334

[B75] Maggiorini M Brunner-La Rocca HP Peth S Fischler M Bohm T Bernheim A Kiencke S Bloch KE Dehnert C Naeije R Lehmann T Bartsch P Mairbaurl H Both tadalafil and dexamethasone may reduce the incidence of high-altitude pulmonary edema: a randomized trial Ann Intern Med 2006 145 497 506 17015867 10.7326/0003-4819-145-7-200610030-00007

[B76] Zimmerman GA Crapo RO Adult respiratory distress syndrome secondary to high altitude pulmonary edema West J Med 1980 133 335 337 7347049 PMC1272327

[B77] Nuckton TJ Alonso JA Kallet RH Daniel BM Pittet JF Eisner MD Matthay MA Pulmonary dead-space fraction as a risk factor for death in the acute respiratory distress syndrome N Engl J Med 2002 346 1281 1286 11973365 10.1056/NEJMoa012835

[B78] Meduri GU Annane D Chrousos GP Marik PE Sinclair SE Activation and regulation of systemic inflammation in ARDS: rationale for prolonged glucocorticoid therapy Chest 2009 136 1631 1643 19801579 10.1378/chest.08-2408

[B79] Lindqvist P Soderberg S Gonzalez MC Tossavainen E Henein MY Echocardiography based estimation of pulmonary vascular resistance in patients with pulmonary hypertension: a simultaneous Doppler echocardiography and cardiac catheterization study Eur J Echocardiogr 2011 12 961 966 22011836 10.1093/ejechocard/jer222

